# A Case of Injury of the Hip by a Fall, with Exfoliation of the Bone

**Published:** 1845-07

**Authors:** John M. Ross

**Affiliations:** Marion, Mississippi


Art. III.—*A Case of Injury of the Hip by a fall, with exfoliation of the Bone.* By John M. Ross, M. D., of Marion, Mississippi.

The case which I propose to detail, if it furnishes nothing new in principle or practice, affords, at least, a striking illustration of the *vis medicatrix naturæ*, and of the aid which it is often in our power to render to her curative efforts, by slight surgical interference.

The subject of the injury and operation which I am about to describe, was a negro man, aged 45 years, of robust frame and good health. Eight years ago he fell from the second story of a house, by which he received a severe contusion in the right hip, but no fracture that could be discovered. The parts were lacerated to a considerable extent, as well as bruised. From bad management, or because the injury was more serious than was suspected at the time, he did not recover from the wound for near a twelve-month. About that time he was able to resume his active occupation, and all that remained of the injury was a dull, dragging pain in the parts that had been wounded, the pain increasing in wet or cloudy weather, as also during any disease. He remained in this condition until Friday, the 13th of March last, when he was suddenly taken with a severe pain in the hip, extending to the lumbar region, accompanied with a severe chill, which was followed by fever. On Saturday the pain extended to the abdomen, and was more severe. The chill and fever also returned. These regular returns continued, with increased pain and soreness, until Tuesday, the 17th, when I was called in great haste to see him.

I found him suffering excrutiating pain, and much prostrated; his countenance was haggard, pupils much dilated; a cold clammy perspiration covered his entire surface. The abdomen was remarkably tender on pressere, and he experienced great difficulty in discharging his urine. It was ascertained that large quantities of pus passed from his bowels; his pulse was about 40 in a minute; he complained of the lower part of his bowels mostly, and said that he could feel something hard there.

From these alarming symptoms, I entered into a close examination, and soon found a spicula of bone lodged in the rectum, so near the external orifice, that I could get hold of it with a common pair of forceps; it was, however, apparently fast and immovable, being held by its rough, serrated edges.

Having the patient in a convenient posture, I made further examination with a probe, and soon found that the bone had not yet passed into the rectum, but was just entering. In size the bone was about one inch and a half in length, and three-quarters of an inch in width, with an irregular, rough appearance. Finding that it could not be removed without an operation, I made an incision by a scalpel, having introduced a grooved director through the anus, and along the superior margin of the bone to its full extent. The cut extended about three-quarters of an inch, and the bone was extracted with a pair of forceps. But little hemorrhage ensued, nor was the quantity of pus discharged as great as I had expected. The operation was completed by bringing the edges of the wound together and applying simple dressings. Afterwards I gave the patient twenty drops of laudanum, and thirty drops of tincture of catechu, and ordered that he should abstain from all highly seasoned or stimulating food and drinks. His pulse soon rose to about 70, and he fell into a tranquil sleep, in which state he remained until the following morning. The next day after the operation, I returned and found him in a state of perfect ease. I ordered him to take two ounces of castor oil, and permitted him to drink a small quantity of soup; directed that he be kept in a perfect state of rest, in a recumbent posture. Returned next day and found my patient quite cheerful, without any discharge of pus, bowels open, skin moist, tongue clean, no appearance of inflammation, divided parts healing well, pulse about 75. I again ordered oil to keep the bowels soluble, and put the patient upon a low diet. I saw him again on the 20th; everything was progressing well; no pain or fever. The same directions to be continued. My visits were now discontinued, and I saw him no more until a few days since, when he had regained his strength, felt his hip stronger than it had been before for 8 years, and with less pain in it, in a word, as he expressed it himself, feeling like a new man.

Marion, April, 1845.

